# Biconically Tapered Fiber Optic Probes for Rapid Label-Free Immunoassays ǂ

**DOI:** 10.3390/bios5020158

**Published:** 2015-04-01

**Authors:** John Miller, Angelica Castaneda, Kun Ho Lee, Martin Sanchez, Adrian Ortiz, Ekrem Almaz, Zuleyha Turkoglu Almaz, Shelton Murinda, Wei-Jen Lin, Ertan Salik

**Affiliations:** 1 Department of Physics and Astronomy, University of California, Los Angeles, 475 Portola Plaza, Los Angeles, CA 90095, USA; E-Mail: johnmiller@physics.ucla.edu; 2 Department of Plant and Microbial Biology, University of California, Berkeley, 565 Li Ka Shing Center, Berkeley, CA 94720, USA; E-Mail: afcastaneda@berkeley.edu; 3 Department of Biological Sciences, California State Polytechnic University, Pomona, 3801 W Temple Ave, Pomona, CA 91768, USA; E-Mails: kunhlee@cpp.edu (K.H.L.); aaortiz@cpp.edu (A.O.); weijenlin@cpp.edu (W.-J.L.); 4 Department of Physics and Astronomy, California State Polytechnic University, Pomona, 3801 W Temple Ave, Pomona, CA 91768, USA; E-Mail: msanchez2@cpp.edu; 5 Department of Physics, Mus Alparslan University, Istasyon Cad. 49100, Mus, Turkey; E-Mail: e.almaz@alparslan.edu.tr; 6 Department of Biology, Mus Alparslan University, Istasyon Cad. 49100, Mus, Turkey; E-Mail: z.turkoglu@alparslan.edu.tr; 7 Department of Animal and Veterinary Sciences, California State Polytechnic University, Pomona, 3801 W Temple Ave, Pomona, CA 91768, USA; E-Mail: semurinda@cpp.edu

**Keywords:** fiber optic biosensor, tapered fiber sensor, biosensor, immunoassay, label-free biosensor, protein detection

## Abstract

We report use of U-shaped biconically tapered optical fibers (BTOF) as probes for label-free immunoassays. The tapered regions of the sensors were functionalized by immobilization of immunoglobulin-G (Ig-G) and tested for detection of anti-IgG at concentrations of 50 ng/mL to 50 µg/mL. Antibody-antigen reaction creates a biological nanolayer modifying the waveguide structure leading to a change in the sensor signal, which allows real-time monitoring. The kinetics of the antibody (mouse Ig-G)-antigen (rabbit anti-mouse IgG) reactions was studied. Hydrofluoric acid treatment makes the sensitive region thinner to enhance sensitivity, which we confirmed by experiments and simulations. The limit of detection for the sensor was estimated to be less than 50 ng/mL. Utilization of the rate of the sensor peak shift within the first few minutes of the antibody-antigen reaction is proposed as a rapid protein detection method.

## 1. Introduction

**Figure 1:**
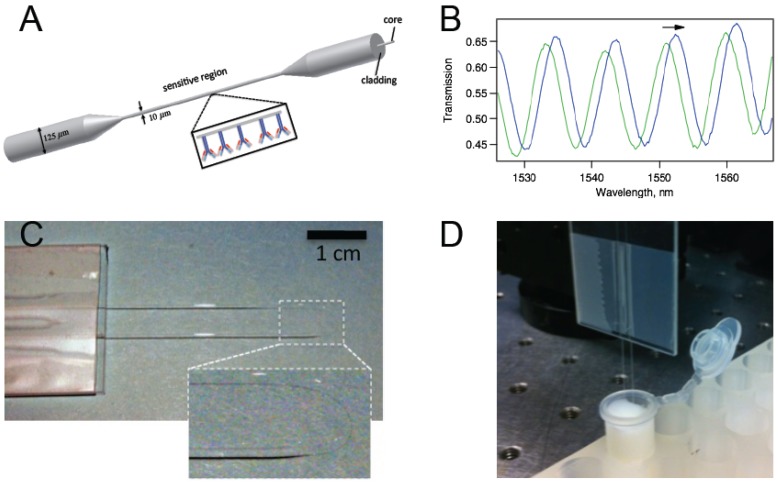
(**a**) A schematic representation of a biconically tapered fiber sensor with antibodies on the surface; (**b**) Interference of the two modes (typically HE11 and HE12) excited in the tapered region gives rise to a transmission spectrum with trackable peaks; (**c**) Photo of a biconically tapered fiber sensor formed into a U-shape. The inset shows a magnified version of the thin region; (**d**) The biosensor dipped in a solution within a test tube.

A biconically tapered optical fiber (BTOF) is a simple and cost-effective refractive index (RI) sensor based on modal Mach-Zehnder interferometry [1,2,3,4]. For biosensing applications, the surface of the taper is typically functionalized with antibodies specific to an antigen to be detected (Figure 1a). The antibody-antigen reactions create a biological nanolayer on the surface of the tapered region that modifies the waveguide structure, which in turn affects the phase difference between the propagation modes. Because the two of the lowest order HE modes are excited, we observe a sinusoidal pattern for the transmission spectrum. (Figure 1b) Spectral shifts due to modifications in the vicinity of the sensor are measured. Unlike biosensors that work based on fluorescent labels, the data can be recorded in real-time enabling the study of the kinetics of antibody–antigen binding. BTOFs in different schemes were demonstrated for detection of bacteria [5], toxins [6], proteins [7,8,9,10], and nucleic acids [11].

In most previous studies, BTOFs based on Mach-Zehnder interferometry were reported for sensors in straight geometry [2,3,4,9]. The straight fiber taper cannot be used as a dip probe that can be immersed in solutions because of geometrical limitations. In addition, when there is a temperature increase, the substrate of the taper expands and the tensile stress on the sensor increases leading to the possibility of breakage and increased temperature sensitivity.

In order to convert our BTOF to a dip probe and avoid any mechanical failure or increased temperature sensitivity, we used a U-shaped geometry in our experiments. (Figure 1c) Although similar U-shaped tapered or non-tapered fiber optic probes were previously studied [12,13,14,15], such U-shaped geometry has not been reported for BTOFs based on modal Mach-Zehnder interferometry. As an added benefit, in the U-shaped geometry, evanescent field penetration increases enhancing detection sensitivity [12].

To demonstrate biosensing, we functionalized U-shaped BTOF with mouse IgG, and tested the sensor functionality by monitoring the kinetics of the mouse IgG and rabbit anti-mouse IgG reaction at concentrations of anti-IgG in the range 50 ng/mL to 50 µg/mL. We investigated the IgG-anti-IgG reaction kinetics, as well as the possibility of monitoring the rate of the reaction for rapid detection. We tested the enhancement of sensitivity through hydrofluoric acid (HF) treatment of our sensors, which we also investigated through computer simulations.

## 2. Experiments

### 2.1. Sensor Fabrication

Biconical tapers were made by exposing single-mode optical fibers (Corning SMF-28) to the flame of a butane torch while they are under tension. The fiber was fixed at both ends, and tension was applied by pulling one end with an actuator. The torch was located at a constant point on the fiber throughout the tapering process. Each fiber was pulled to a length of 2.0 cm, producing a taper waist region of 1.5 ± 0.1 cm in length, and 10 ± 1 µm in diameter, as confirmed with an optical microscope. The sensor was formed in a U-shape after fabrication and affixed to a glass slide. Figure 1c,d display pictures of the U-shaped sensor.

Because we found that sensors with thinner taper waists became more sensitive, we also subjected some sensors to hydrofluoric acid (HF) treatment by incubating them in 49% HF at room temperature for a few minutes. The taper waist diameter was reduced to <5 µm. Reduced thickness modifies the mode propagation constants, which leads to increased sensitivity.

### 2.2. Reagents and Antibodies

The protein crosslinking chemicals 3-APTES (3-aminopropyltriethoxysilane) (#80370), EDC (1-Ethyl-3-[3-dimethylaminopropyl]carbodiimide Hydrochloride) (#22980), and NHS (*N*-hydroxysuccinimide) (#24500) were purchased from Thermo Fisher Scientific (Rockford, IL, USA). BSA (bovine serum albumin) (#A7906), BME (b-Mercaptoethanol) (#M6250), MES (2-[N-Morpholino]ethanesulfonic acid) (#M3671), and NaCl (Sodium chloride) (#S3014) were purchased from Sigma Aldrich (St. Louis, MO, USA). Tween-20 (#BP 337-100) was purchased from Fisher Scientific (Fair Lawn, NJ, USA). Na_2_HPO_4_ (Sodium phosphate dibasic) (#7782856) was purchased from Acros Organics (NJ, USA). KCl (Potassium chloride) (#7300) was purchased from Merck KGaA (Darmstadt, Germany). KH_2_PO_4_ (Potassium phosphate monobasic) (#53246) was purchased from J.T. Baker Chemical Co (Phillipsburg, NJ, USA). The antibody rabbit anti-mouse IgG (#31194) was purchased from Thermo Fisher Scientific. Purified Mouse IgG (#026502) was purchased from Invitrogen (Frederick, MD, USA). BS^3^ (bis[sulfosuccinimidyl] suberate) (#21580) and anhydrous ethanol were purchased from Thermo Fisher Scientific. Hydrofluoric Acid (HF) (#423805000) was purchased from Acron Organics.

### 2.3. Protein Immobilization on the Sensor Surface 

For protein crosslinking by the EDC method [7], the sensors were sequentially treated with plasma for 2 min followed by incubation in 1% APTES in ultra pure water, pH 3.0, for 2 h at 75 °C. Mouse IgG was pre-treated with 2 mM EDC and 5 mM NHS in activation buffer (0.1 M MES and 0.5 M NaCl, pH 6.0) for 15 min at room temperature, after which the EDC reaction was quenched with BME to a final concentration of 20 mM. The EDC-conjugated IgG complex was reacted with the sensor’s activated surface for 2 h at room temperature (RT). Following protein immobilization, the sensor was incubated overnight in 3% BSA in PBST (1× phosphate buffered saline with 0.05% Tween) to block nonspecific binding during testing.

We used an alternative protocol for protein immobilization. This protocol started with plasma treatment of the sensor surface, followed by 30 min incubation in 5% 3-APTES in anhydrous ethanol at RT. After an ethanol wash, the sensor was treated with 5 mM BS3 in PBS for 60 min at RT. The sensor was washed in PBS, and incubation in 500 µg/mL IgG in PBS for 1–2 h at RT. Finally, the sensor was incubated in 500 µg/mL BSA in PBST (1× phosphate buffered saline with 0.05% Tween) at 4 °C at least 12 h.

The antibody to be detected, rabbit anti-mouse IgG, was prepared in PBST. A solution of PBST with BSA at the same concentration as the target protein was used as a negative control.

### 2.4. Testing the Sensor 

**Figure 2:**
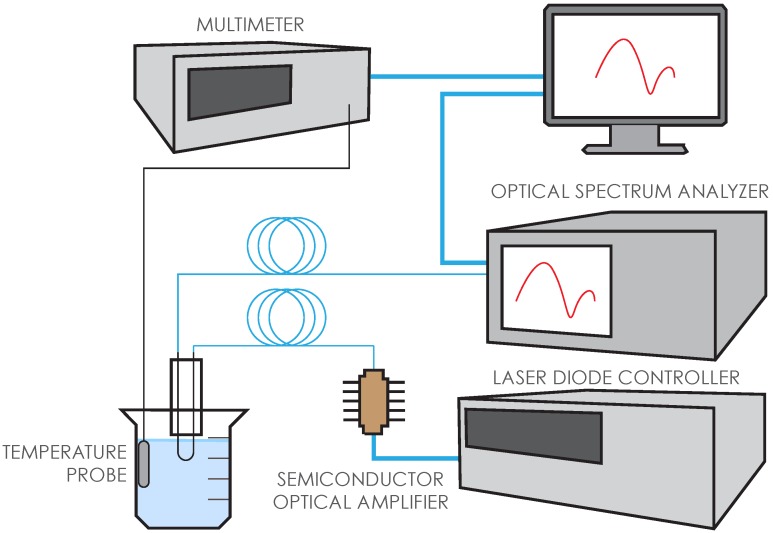
Sensor interrogation system. Amplified spontaneous emission from the semiconductor optical amplifier is used as the broadband light source in the 1450–1600 nm wavelength range. Data acquired from the optical spectrum analyzer was saved in a PC using Labview.

Figure 2 shows our setup for testing the functionalized sensors. Amplified spontaneous emission from a semiconductor optical amplifier (SOA) was used as a broadband light source (1450–1600 nm). A 1 × 5 optical switch allowed us to measure the source and the sensor response ~5 s apart and up to 4 different sensors can be automatically interrogated with one channel reserved for reference measurement to account for any fluctuations in the source power. In actual tests, we usually interrogated three sensors simultaneously while immersed in each solution. Every ~20 s a sensor spectrum was recorded. Five-minute immersions in PBST served as baseline measurements between all other solutions. Fifteen-minute immersions in BSA/PBST served as a negative control for non-specific binding, both before and after testing in anti-IgG. The sensing of the target protein (anti-IgG) to the IgG-coated sensors was measured for at least 30 min. Throughout the entire measurement, the solutions were kept within a solid aluminum heat block that was immersed in a water bath to keep the temperature stable. A temperature probe, which was kept adjacent to the heat block within the water bath, was used to monitor temperature for every measurement. In addition to all control measurements, we collected the sensor transmission spectra for at least 1 h in the wavelength range of 1475–1575 nm with resolution at 1.0 nm, which amounts to at least 200 spectra to be analyzed.

### 2.5. Data Analysis 

**Figure 3:**
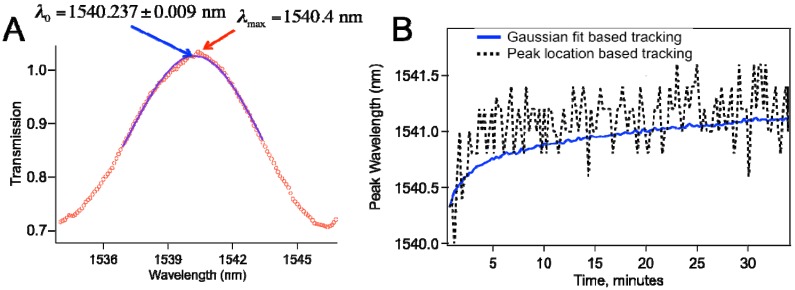
(**a**) Gaussian fitting to the data leads to determination of the peak with better precision; (**b**) Comparison of the peak wavelength *vs.* time plot based on Gaussian fit based tracking and that with peak location search based tracking.

As Figure 1b shows that the sensor spectra involved multiple peaks with about 5–10 nm full width at half maximum (FWHM). To determine the peak shift, we wrote a script in Igor Pro (Wavemetrics, Portland, OR, USA) that finds a peak by a Gaussian fit to the spectra around a local peak. The script first finds the wavelength for the local maximum (*λ*_c_) and then runs the Gaussian fit between *λ*_c_ − FWHM/2 and λ_c_ + FWHM/2 similar to what was reported for Fiber Bragg Gratings [16]. Specifically, we fitted to the following function:

(1) $$T=T_{0}+A\exp\left[-{\left(\frac{\lambda -\lambda_{0}}{w}\right)}^{2}\right]$$

where λ_0_ represents the peak center wavelength, which is the desired fit parameter. Because of the inherent noise in the system as well as the digitization error of the OSA, Gaussian fitting based tracking proves very useful, as illustrated in Figure 3. In our typical spectra, we have 1000 points, and we typically use 100-nm wavelength range. As shown in Figure 3a, we can determine the peak by maximum searching to the first digit, and have slight unavoidable shifts due to noise. Figure 3b compares peak wavelength data obtained through Gaussian fit based tracking with maximum location search based tracking.

### 2.6. Simulations 

To study our sensors using computer simulations, we first calculated the effective refractive index (and the propagation constant *β*) of the propogation modes in a step-index fiber by numerically solving the following eigenvalue equation [17]:

(2) $$\left[\frac{J_{1}^{\prime }\left(u\right)}{uJ_{1}\left(u\right)}+\frac{K_{1}^{\prime }\left(w\right)}{wK_{1}\left(w\right)}\right]\left[\frac{J_{1}^{\prime }\left(u\right)}{uJ_{1}\left(u\right)}+\frac{n_{cl}^{2}K_{1}^{\prime }\left(w\right)}{n_{co}^{2}wK_{1}\left(w\right)}\right]={\left(\frac{n_{eff}}{n_{co}}\frac{V^{2}}{u^{2}w^{2}}\right)}^{2}$$

where *n_co_* and *n_cl_* are the refractive index of the core and the cladding respectively. *n_eff_* is the effective refractive index of the propagation mode, and *J_i_* and *K_i_* are the Bessel functions of the first and second kind. The parameters *u*, *w*, and *V* are given by:

(3) $$\begin{array}{l} u=\frac{2\pi }{\lambda }r\sqrt{n_{co}^{2}-n_{eff}^{2}}\\ w=\frac{2\pi }{\lambda }r\sqrt{n_{eff}^{2}-n_{cl}^{2}}\\ V=\frac{2\pi }{\lambda }r\sqrt{n_{co}^{2}-n_{cl}^{2}} \end{array}$$

where *r* is the radius, and *λ* is the wavelength in vacuum.

When there is a nanolayer of thickness δ*r* and refractive index of *n_lyr_* between the core and the cladding; an approximate solution for the propagation constant of the modes is possible [17]:

(4) $$\beta^{\prime }=\beta +\frac{4\pi }{\lambda }\left(n_{lyr}-n_{cl}\right)\frac{\delta r}{r}{\left[\frac{u}{V}\frac{K_{0}\left(w\right)}{K_{1}\left(w\right)}\right]}^{2}$$

where *β*′ and *β* represent the propagation constants with and without the deposited nanolayer on the sensor surface. For our biosensors, the nanolayer deposited on the surface consists of proteins.

Transmission spectra through the tapered fiber sensor can be calculated by a simple interference equation as typically two modes of propagation are excited in the taper waist. We use:

(5) $$P_{out}=P_{1}+P_{2}+2\sqrt{P_{1}P_{2}}\cos\left(\text{Δ}\phi \right)$$

where *P*_1_ and *P*_2_ are the optical powers coupled to the HE_11_ and HE_12_ modes, respectively. Δ*ϕ* is the phase difference between the modes.

## 3. Results and Discussion

**Figure 4:**
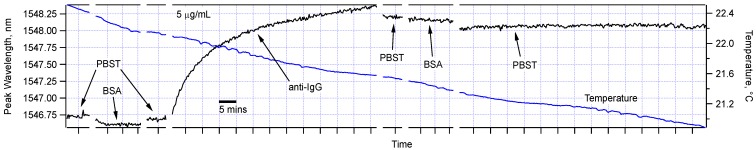
The functionalized sensor was sequentially immersed in PBST, BSA (5 µg/mL), PBST, anti-IgG (5 µg/mL), PBST, BSA (5 µg/mL), and PBST. As indicated by the scale bar, each horizontal division is 5 min. Temperature variation during of the water bath was also simultaneously recorded during the experiment and was shown here.

Figure 4 shows the peak wavelengths of the full sequence of measurements for one of the sensors. It demonstrates that while control measurements in PBST and BSA solutions did not cause any significant shift to the sensor peaks, the sensor transmission spectrum was significantly modified in the solution containing the target protein, anti-IgG. The measurements in PBST and BSA after the anti-IgG step confirm that the shift is specific and permanent indicating the formation of a nano-scale biological layer on the sensor surface. Notably, we recorded the sensor spectrum for almost 1 h in PBST with no peak shift, so we can state that the IgG-anti-IgG affinity binding were in effect, and there was no physical adsorption mechanism except for a slight shift immediately after the anti-IgG test. We also observed very small temperature sensitivity throughout this experiment. Particularly, in the last PBST step, the temperature around the sensor changed by about 0.5 °C, but there was a shift of no more than 20 picometers (pm). This is indeed what is expected as verified by simulation using the data for refractive indices of water and glass as a function of temperature [18,19,20] and Mathematica (Wolfram Research, Champaign, IL). At room temperature, the thermo-optic coefficient for water and fused silica are about 8 × 10^−5^ RIU/°C and 1 × 10^−5^ RIU/°C, respectively. Using these values we calculated the temperature sensitivity of our sensors to be about 15 pm/°C for a sensor ~10 µm in diameter and 1.5 cm in length.

**Figure 5:**
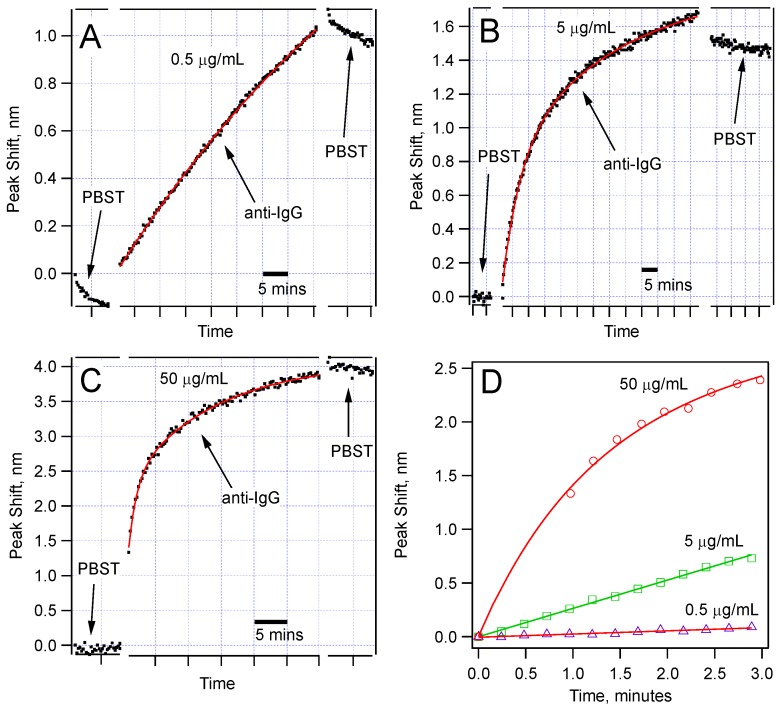
Sensor response for three different concentrations of anti-IgG: (**a**) 0.5 µg/mL; (**b**) 5.0 µg/mL; (**c**) 50 µg/mL; Note that different wavelength scales are used to show the details of the dependence. The time scale is expressed using scale bars. The data fit best to double exponential functions; (**d**) Comparison of the peak shift in the first few minutes of the anti-IgG binding showing different rates for different concentrations.

Figure 5 shows the comparison of the spectral shifts with time for three concentrations (0.5, 5, and 50 µg/mL) of the anti-IgG solution. Each of these sensors was monitored for at least 30 min during IgG-anti-IgG binding. All of them show permanent peak shifts as a result of IgG-anti-IgG binding. For the 50 µg/mL anti-IgG test, we observed a very sharp shift right after the sensor was immersed in the anti-IgG solution. Given that we can collect a data set once in every 20 s, we could not capture the initial data points. We expect this jump is due to very rapid binding at the high IgG concentration. But the sudden change in the average refractive index is also worth analyzing. It is well established that the refractive index of protein solutions is proportional to concentration:

(6) $$n_{ps}=n_{s}+a\times C$$

where *n*_ps_ and *n*_s_ are the refractive indices of the protein solution and the base solution, respectively [21]. If *C* is the concentration of the protein in grams/(100 mL), then the proportionality constant *a* becomes about 2 × 10^−3^ mL/g. Using this relationship, we compute the change in refractive index (*n*_ps_ − *n*_s_) to be ~10^−5^, which is expected to cause a peak shift of <0.1 nm. However, we measured a ~1.3 nm sudden shift. This is very hard to explain by mere average refractive index change around the sensor. Therefore, a permanent protein nanolayer should be forming on the sensor surface extremely quickly. The measurements in PBST solutions before and after the IgG-anti-IgG binding stage also confirm this conclusion. Therefore, the sensing mechanism is a surface mechanism, as expected of biosensors.

Another feature that we noticed was a slight downshift observed in some cases, for example in PBST solutions for 0.5 µg/mL, and a similar one was observed in the PBST after the 5 μg/mL anti-IgG incubation. We propose that such downshifts are due to the release of some physically adsorbed proteins. Removal of the proteins from the surface is expected to reduce the effective nano-layer thickness and give a negative shift of the sensor peaks.

Figure 5d compares the rate of the peak shift in the first 3 min of the binding experiments. The data show a concentration-dependent response. This comparison suggests that using the data in the first few minutes, one can make a prediction about the concentration of the target protein in a solution. Obviously, for this method to be broadly applicable, we should further study the reaction kinetics when the sensor is tested for detection of a target protein in the presence of other proteins, or in native environments of interest, such as blood serum or milk. Toward this goal, we have validated detection of anti-IgG of varying concentrations (500 ng/mL to 5 µg/mL) in a solution with high concentration of BSA (500 µg/mL). Our results were identical to the ones obtained in pure anti-IgG solutions.

**Figure 6:**
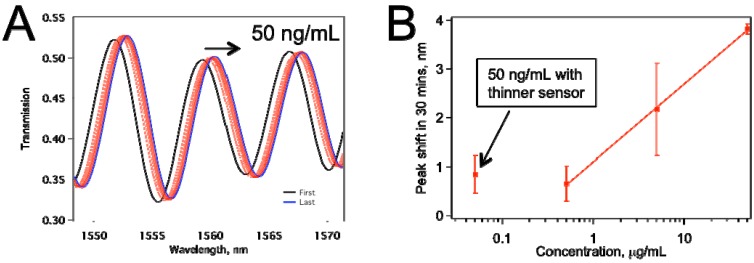
(**a**) Peak shift for a tapered fiber sensor that was HF treated. The anti-IgG concentration was 50 ng/mL; (**b**) The comparison of the thinner sensor performance with sensors ~10 µm thickness. Each data point represents at least two separate experiments.

In an effort to enhance the sensitivity of our sensors, so that we can detect anti-IgG concentrations lower than 500 ng/mL, we subjected our U-shaped sensors to HF treatment, as explained in Section 2.1. Our refractive index sensing tests with ethanol-water mixtures showed that the sensors became ~5 times more sensitive in comparison with the non-HF treated ones. Figure 6 displays data for a biosensor that was treated with HF to enhance its sensitivity, as explained in Section 2.1. This sensor was thinner because of the etching by HF. Figure 6a shows the raw data for the peaks shifting to the right as the IgG on the sensor surface binding with anti-IgG. Figure 6b summarizes peak shifts for various concentrations tested. The sensors tested for 0.5, 5.0, 50.0 µg/mL were about 10 µm thickness, whereas the ones tested for 50 ng/mL anti-IgG were treated with HF, and they were thinner. The comparison confirms our expectation that the thinner sensor provided better limit of detection (LOD), and the average peak shift obtained at 50 ng/mL is about the same as that with 500 ng/mL when thicker sensors were used. Also, we have not observed an increase in the frequency of mechanical failure in the HF-etched sensors in comparison with non-etched ones.

**Figure 7:**
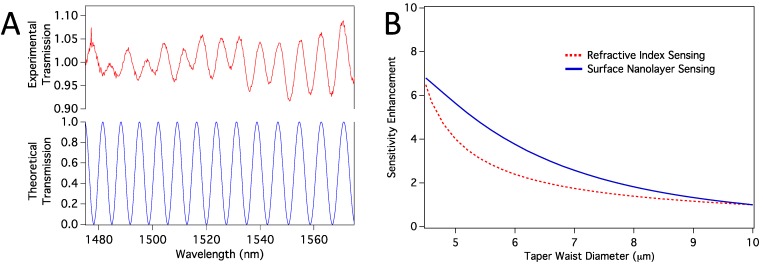
(**a**) Comparison of experimental and theoretical transmission spectra; (**b**) Sensitivity enhancement relative to the sensitivity of a sensor with taper waist diameter of 10 µm. Both volume RI sensing and surface nanolayer sensing cases are shown.

Enhanced sensitivity of the HF-etched sensors should be mainly because HF etching makes the taper waist thinner, and the difference between the phases of the propagation modes increase more rapidly with any change in the vicinity of the sensor surface. In order to confirm this explanation, we have conducted computer simulations, as explained in Section 2.6. We first confirmed that the propagation modes excited in the tapered region are HE11 and HE12 modes. When we calculated the transmission spectra, we found them closely resemble the experimental spectra, as shown in Figure 7a. Note that, the number and location of the peaks are confirmed with the simulations, and the absolute experimental transmission depends on mode coupling strength, which we have not considered. However, because the sensor works based on the peak shifts, mode-coupling strength is not relevant. We then calculated sensitivity enhancement relative to a sensor with 10 µm taper waist diameter. Sensitivity here means how much peak shift happens in response to a change in the refractive index of the medium around the taper waist. We considered both volume refractive index sensing and surface sensing cases. In surface sensing, we assumed a protein nanolayer of 10 nm thickness to be deposited on the sensor surface while the average refractive index of the bulk medium stays the same. Figure 7b shows calculated sensitivity enhancements for both volume RI sensing and the surface protein layer sensing. In both cases, we expect enhancement as we reduce the thickness from about 10 µm to <5 µm.

The kinetics of the entire process is also interesting. IgG anti-IgG binding kinetics was studied in 1974 using nephelometry [22]. Later this was repeated using an immuno-precipitation approach where the bound and unbound antigens were measured using a ^125^I- labeled antigen [23]. The study found a linear relationship of mouse anti-human IgG binding to human IgG1 kappa antigen. More recently, surface plasmon resonance was also used to measure such kinetics [24]. Our method offers an alternative way to measure the IgG anti-IgG binding kinetics. One key difference between our study and the previous reports is the use of a cylindrical optical fiber surface, as opposed to flat or spherical surfaces.

**Figure 8:**
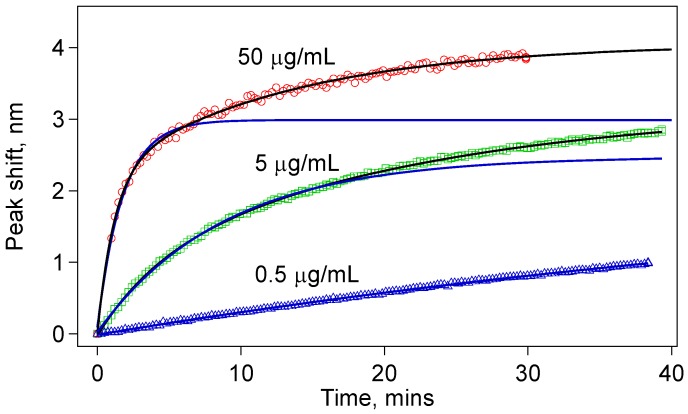
The data for the tapered fiber sensors do not fit to a simple exponential (blue solid curves). They rather fit to a double exponential indicating two rate constants. This behavior is especially clear with higher concentrations.

A single IgG molecule on the sensor surface binds with a single anti-IgG molecule in the solution to give a single bound complex contributing the effective thickness of the biological nanolayer on the sensor surface. This suggests first-degree reaction kinetics, which is expected to give an exponential response. However, as shown in Figure 7, for especially 5 µg/mL and 50 µg/mL, we found that the data fit better to a double exponential function given by:

(7) $$\text{Δ}\lambda =\text{Δ}\lambda_{0}+A_{1}e^{-\left(\frac{t-t_{0}}{\tau_{1}}\right)}+A_{2}e^{-\left(\frac{t-t_{0}}{\tau_{2}}\right)}$$

where τ_1_ and τ_2_ are the two separate time constants for each process. The peak shift, Δλ, should be understood as an indicator of the anti-IgG layer thickness on the sensor surface. Before any antibody–antigen binding occurs, there is a finite number of antibody binding sites on the sensor surface. Depending on the concentration, we observe a fast binding process initially. After a while, many antibody-binding sites are already populated, and the probability of an unbound antigen binding one of the antibody sites decreases significantly. However, we do not see a saturation of this fast initial exponential process, which are shown by blue curves in Figure 8. Rather, the binding events continue at a slower rate indicative of the double-exponential fit function given in Equation (7).

The double-exponential response we noted was also observed for the IgG-anti-IgG binding measured by surface plasmon resonance [24], which is most probably because the reaction happens on a surface rather than within a uniform mixture of molecules in a solution. The anti-IgG molecules need to be transported to the sensor surface before any reaction can happen, which becomes especially important after the initial IgG sites are populated [25]. The transport-limitation here should not just be a simple diffusion-related event, as the protein diffusion is fast. A protein of 5 nm in size is expected to move about 0.5 µm in 1 ms [26]. Possibly, some of the free and bound molecules prevent other free molecules from reacting with existing antibody sites [27].

Finally, based on our analysis of the data we can claim a LOD lower than 50 ng/mL for IgG-anti-IgG binding. Considering that we can measure 10 pm shifts using our OSA and the peak finding algorithm based on Gaussian fitting, we can claim that the LOD is lower than 50 ng/mL. This LOD is comparable to surface plasmon resonance-based detection [24], and it is within the range of typical conventional immunoassays. For rapid detection, we can utilize the rate of the peak shift in the first 3 min. We measured an average peak shift of 380 pm at 50 ng/mL within 3 min of the start of the test, which is still significantly higher than 10 pm limit of our measurement platform. For even lower concentrations, the detection time can be somewhat longer than 3 min to better predict the concentration.

## 4. Conclusions

In conclusion, we have demonstrated BTOF in a U-shape as a more robust biosensor in comparison with the straight version. The sensor is able to detect, in real time, the binding of the target protein to the surface of BTOF through Ab-Ag interactions in a concentration-dependent manner. The concentrations we tested in this study go as low as 50 ng/mL, which is as sensitive as a typical immunoassay. Using a simple and controlled buffering system, we have demonstrated, as proof-of-concept, that the BTOF in a U-shape configuration is able to work as a biosensor. Analysis of the data showed that IgG-anti-IgG binding has at least two processes governing the reaction kinetics. For a given sensor geometry, we found relatively good correlation between peak shift for the sensor and the concentration (Figure 5d and Figure 6b), which suggests the ability to predict concentration of the protein rapidly. The initial fast reaction rate can be used to estimate the protein concentration within 3 min of monitoring the IgG-anti-IgG binding.

Future studies will include determination of the detection limit of the sensor, especially in complex environments such as blood serum or milk focusing on validation of protein concentration measurement using only data collected in the first few minutes. To make the sensor very robust, our development for embedding the sensor within microfluidic channels is also underway. HF treatment to make the sensor thinner enabled us to make the sensors more sensitive, and we will work to optimize the sensor geometry for the best sensitivity.
